# Social Deficits or Interactional Differences? Interrogating Perspectives on Social Functioning in Autism

**DOI:** 10.3389/fpsyt.2022.823736

**Published:** 2022-04-25

**Authors:** Xiangting Bernice Lin, Choon Guan Lim, Tih-Shih Lee

**Affiliations:** ^1^Neuroscience and Behavioral Disorders Program, Duke-NUS Medical School, Singapore, Singapore; ^2^School of Social Sciences, Nanyang Technological University, Singapore, Singapore; ^3^Department of Child and Adolescent Psychiatry, Institute of Mental Health, Singapore, Singapore; ^4^Department of Psychiatry, Singapore General Hospital, Singapore, Singapore

**Keywords:** autism spectrum disorder, social functioning, review, neurodiversity, neurodevelopmental conditions

## Abstract

Social dysfunction is a key characteristic of autism. Determining and treating autism-related social deficits have been challenging. The medical model views interpersonal difficulties in autism as a localized set of deficits to be managed, whereas the neurodiversity movement calls for the accommodation of differences by the larger community. One common assumption underlying these perspectives is a misalignment in social behaviors between autistic individuals and neurotypicals. This paper reviews and interrogates current perspectives on social functioning in autism to uncover the intricacies of such a notion. Even though extant literature has alluded to a misalignment in social behaviors between autistic and neurotypical individuals, it is uncertain where this disparity lies. Implications for future research and practice are discussed.

## Introduction

Autism Spectrum Disorder (ASD) is a heterogenous group of neurodevelopmental conditions characterized by social dysfunction and restricted, stereotyped behaviors [Diagnostic and Statistical Manual of Mental Disorders, 5th Edition (DSM-5); (1)]. Social deficits range from lack of social-emotional reciprocity and poor nonverbal communication to difficulties in developing and maintaining relationships. Comorbidities such as intellectual disability and anxiety disorders are common (2–5). Worldwide prevalence estimates of ASD are highly variable and rising, i.e., 0.08 – 9.3% (6). About one in 54 children in the United States were identified with ASD, with ASD being 4.3 times more common in boys than in girls (7). Overall prevalence rate of ASD is estimated at 0.36% across Asia (8).

Individuals living with ASD have substantial mental healthcare needs. Psychiatric service utilization and use of psychotropic medications to manage comorbid psychiatric conditions are high (9). Notably, there is no cure at present for core ASD symptoms of social deficits and stereotyped behaviors. Early structured and targeted behavioral interventions may be helpful only to reduce these symptoms and maximize capacities for daily functioning (10). Besides, ASD has been associated with significant healthcare burden. Several studies have reported on the high direct and indirect costs of managing the disorder (11–14). Disability-adjusted life-years for ASD have increased steadily from 1990 to 2016 (15), indicating a growing burden and poorer quality of life among sufferers.

Issues regarding ASD are exacerbated by an evolving nosology that remains fraught with longstanding difficulties. Problems with characterizing ASD have been largely attributed to the lack of a single known etiological pathway, a behavioral focus in diagnosis, and heterogenous phenotypic or behavioral manifestations. At present, ASD is understood to involve multiple possible factors that lead to an arguably similar behavioral outcome (16). Still, no one individual with ASD behaves the same way as another. If no single set of etiological factors or behavioral outcomes defines ASD, what makes an individual autistic? How does the clinician conclude based on reported or observed behaviors that one has ASD? Existing measurement tools are inadequate in discriminating marginal cases or translating quantitative outcomes to clinical diagnosis (17). Accurate diagnoses stay hampered by variability in clinical characteristics and limited access to multidisciplinary assessments (18). Besides, differential prognoses affect the utility of an ASD diagnosis (19). Even though theories and models have been proposed to account for its etiology (20, 21), they remain unintegrated, contentious, or subject to further validation.

Notwithstanding the enduring backdrop of challenges, interpersonal communication difficulties remain a central feature of ASD. Recently, the neurodiversity movement, which opposes the deficit-based medical model and propounds the view that ASD and other neurological impairments are normal human differences, has gained traction. This approach argues for a shift from treatment to an acceptance of characteristic differences between ASD and neurotypicals. Gillespie-Lynch et al. (22) conducted a survey involving both autistic and non-autistic persons. They found that autistic individuals perceived less importance in finding a cure and were inclined to view ASD as a biological difference rather than a deficit. However, the distinction between ASD-as-difference and ASD-as-deficit appears to be more complex: autistic and non-autistic persons did not differ in terms of negative emotions about and having support for ASD (23). Consequently, a deficit-as-difference model that acknowledges overlaps between the medical paradigm and neurodiverse view has been proposed.

While the medical model and neurodiversity movement seem to contend with how ASD should be addressed, a closer look suggests that these two approaches operate at disparate societal levels. The medical model responds to ASD as a localized set of deficits to be cured or managed, whereas the neurodiversity movement calls for ASD to be accommodated by the larger community. Regardless, one common assumption is a misalignment in social behaviors between autistic individuals and neurotypicals. This paper reviews and interrogates current perspectives on social functioning in ASD to uncover the intricacies of such a notion.

## Perspectives From Neuroscience and Biology

Biological and genetics research in ASD have centered primarily on establishing genetic links to or risk factors for ASD and related traits. Genome-wide association studies found ASD to significantly correlate with deleterious de novo mutations (24, 25). Heritability has been indicated in family studies investigating unaffected relatives' genetic liability to ASD-related social characteristics, such as language irregularities, aloofness, rigidity, hypersensitivity to criticism, and reduced number and quality of friendships (26–28). Animal models help dissect the specific roles of genetic and environmental factors in ASD pathogenesis, untangle the relationships between social behavior and altered genes, genetic expression, or brain anatomy and function, as well as provide a means to test effects of pharmacotherapy (29–31). A range of animal models including mouse, flies, and primates has been used to improve translational outcomes (32, 33). While they remain highly integral to establishing links between ASD phenotypes and genetic, epigenetic, environmental, and neurobiochemical factors, these studies have assumed ASD a priori or investigated aspects of social functioning on proxy measures or species. Applications of drug discoveries through animal models are challenged by small and heterogeneous human clinical samples (34). How findings translate to the actual interpersonal functioning of individuals with ASD are subject to cautious interpretations.

Neuroimaging and behavioral studies complement animal studies and reveal added insights into the interpersonal deficits or functioning among autistic persons. In general, such efforts support the view that core social deficits are associated with differences in the brain or cognitive function. These include structural and functional abnormalities in “social brain” correlates such as the cerebellum, amygdala, inferior frontal gyrus, and ventromedial prefrontal cortex (35–37). In the examination of higher order social cognitive processes, ASD social deficits have also been linked to variabilities in joint attention, social orienting, theory of mind, empathy, and eye gaze behavior (38–44). Critically, the “social brain” that supports these social cognitive processes is more complex than what has been described so far. It implicates a distributed collection of neural networks and a highly intricate, multilevel neurobiochemical process that influence perception, emotion, motivation, and executive function (45). Abnormalities can occur at any point or neural circuit in this process, ranging from neuroanatomical and genetic aberrations to deficits in the oxytocin or dopaminergic systems, yet contribute to a similar ASD phenotypic profile (46). The “social brain” largely overlaps with the brain reward circuit, but isolating the neural circuitry or variants relevant to ASD-related social deficits has been challenging.

Overall evidence in neuroimaging and behavioral studies are mixed. Guillon et al. (47) conducted a review of eye-tracking studies and failed to find consistent support for deficits in social orienting or eye gaze in ASD. Neuroimaging outcomes of ASD have also not been replicated, as ASD plausibly implicates “several large-scale neurocognitive networks” that are yet unknown (48). Intranasal oxytocin that has been widely advocated to modulate neural mechanisms and thus improve social behaviors in ASD is found ineffective alone and is likely only effective when implemented in appropriate contexts, such as alongside behavioral therapy (49). Furthermore, clinical neuroimaging has only been performed in high functioning ASD patients, for which FMRI scanning was acceptable. Heterogeneity in age, phenotypic expression of ASD, psychotropic medications, and/ or participation in behavioral therapy programs constitute another major source of bias in these neuroimaging studies.

Advances in neuroscience and biology have shown that ASD is not a result of single gene or a fixed constellation of genetic or neurological differences. Constantino (50) articulated the complexities involving genetic bases and phenotypic expression of ASD, positing that ASD is an aggregation of multiple early behavioral susceptibilities with a genetic basis. Neuroimaging and behavioral studies also found evidence for the heterogenous nature of brain anatomy and cognitive function in ASD across the developmental lifespan (51, 52). Curiously, interactions between cognition and socially relevant factors are intricate and variable. For instance, executive function accounted for theory of mind but not verbal communication in children with ASD (53). Mazefsky et al. (54) examined first-degree relatives of individuals with high functioning ASD and found that family history of shyness and depression predicted autistic persons' adaptive and socialization behavior. Determining ASD-specific or non-specific social behavior, including whether social functioning in ASD is poor, requires a sophisticated analysis of dynamic multilevel factors.

Research on the broad autism phenotype show that core ASD features lie on a continuum and can be observed in neurotypicals (55, 56). Accordingly, even if social deficits can be defined, they are neither a sufficient nor an exclusive feature of ASD. It is worth noting that other psychiatric diagnoses also entail difficulties in social interaction (57–60). What constitutes social dysfunction? What part of it makes individuals autistic? Extant neuroscientific evidence reveals the convolutedness, not the essence, of social functioning in ASD. They have worked from the premise, rather than show, that social functioning in autistic persons is misaligned or problematic.

## Perspectives From Intervention Research

Broadly, treatment for ASD builds upon the neurobiological underpinnings of ASD. Pharmacological treatments for ASD have been proposed based on functional hypotheses or the repurposing of existing compounds through an empirical approach. Given the prevailing lack of agreement in etiology, there is no pharmacotherapy that effectively addresses core ASD challenges. Medicine for ASD is often for commonly co-occurring symptoms apart from social functioning. For example, risperidone and aripiprazole have been used to mitigate agitation and irritability in ASD (61, 62). Even though these symptoms likely interfere with positive social interactions, suppressing challenging behaviors does not necessarily equate to improved interpersonal functioning. Little is known about the relationships between aberrant behaviors and interpersonal difficulties. Where core ASD deficits were examined, there is little to no evidence to suggest that pharmacotherapy alleviates difficulties in social interactions (63, 64). Unlike behavioral symptoms like agitation, social functioning is conceivably more than a single behavioral problem to be tackled. Social challenges have also been assumed a priori. Until the neural substrates of ASD-related social deficits are identified, it remains uncertain whether pharmacotherapy targeting these substrates would be efficient.

Perhaps unsurprisingly then, behavioral therapies have been propounded as the mainstay treatment to minimize ASD-specific social challenges for better independent living. These therapies target functional aspects of social life, including self-expression, emotional awareness, and social problem-solving. Applied behavior analysis and cognitive behavior therapy have shown to improve socially relevant behaviors through reinforcement and/ or explicit skills training (65–67). While interventions have brought forth favorable changes in social functioning, it is unclear which strategies or mechanisms were responsible (68). Neuroimaging studies that investigated the neural mechanisms implicated in treatment response demonstrate variable neural responses to pivotal response treatment [i.e., increased activation in the reward system for those exhibiting hypoactivation vs. decreased activation in subcortical regions for those exhibiting hyperactivation; (69)], as well as variable improvements in EEG activity toward social vis-à-vis nonsocial stimuli between the Early Start Denver Model and a community intervention (70).

While studies that complement intervention evaluation with neuroimaging methods are promising (71), behavioral interventions encompass complex exchanges among participants, therapists, setting, expectations, strategies, intellectual capacity, and actual interactions, which have been difficult to tease apart. Where interventions were targeted, improvements on pertinent behaviors were mixed (72, 73). Interventions may have been recommended to encourage adaptive interpersonal behaviors in ASD, but methodological constraints limit inferences that can be drawn regarding improving social dynamics outside of therapy. To this end, novel early intervention programs in more “naturalistic” conditions, such as parent-implemented programs (74, 75), have been developed to facilitate generalization of outcomes to everyday life.

Although behavioral interventions approach ASD as prima facie set of social deficits to be addressed, not all methods appear to locate social dysfunction within the autistic individual. Many studies have recognized the importance of involving those closely associated with autistic persons in intervention (76–78), suggesting that social dysfunction in ASD implicates unaffected immediate others. This intersubjective locus of social functioning corroborates with how therapist-client relationships affect ASD treatment outcomes (79, 80). It is also consistent with findings on animal-assisted occupational therapies that indicate differential interactional preferences among individuals with ASD (81–83). Contrary to conventional belief regarding social behavior in ASD, autistic persons were found able to interact positively with animals. It is thus conceivable that social dysfunction is not an insular characteristic of ASD but a situated and dynamic inter-person problem implicating both autistic and non-autistic individuals.

The situated perspective of social functioning is underscored in implementation of school-based programs for ASD. ASD is often diagnosed early in children, and schools are children's major social participation apart from familial homes. In an elaborate five-year social skills training project, Crawford et al. (84) found success involving autistic youths, their typically developing peers, parents, and teachers. This School/Community/Home intervention model purports that ASD social behaviors can only be normalized by surrounding them with neurotypical peers in the same context wherein the behaviors were to be enacted. Therefore, intervention efforts have focused on inculcating autistic persons with adaptive interpersonal skills and expanding their networks with non-autistic peers in the school environment. School-based interventions have also been delivered to younger children, albeit highly individualized with video models of neurotypical peers (85).

While outcomes on social initiation, response, and interaction have been laudable (86), studies involving school-based interventions evaluated small sample sizes, were typically led by researchers, and were variable in components and degree of peer engagement. Participants with ASD were also high functioning or without cognitive deficits. When implications of intellectual capacity were examined, cognitive ability and not ASD influenced adaptive outcomes in real-life settings (87). These limit generalizability of findings to all autistic individuals as well as inferences that can be made on the eventual effectiveness of peer-mediated or school-based programs run by staff. In addition, adaptive social functioning in ASD appears to require sustained relational engagement, adequate intellectual capacity, and supportive interpersonal contexts. Explicating how these factors align social dynamics or preferences seems crucial to understanding social dysfunction in ASD, as well as to developing effective ASD interventions.

The ambiguity surrounding ASD etiology, diagnostics, and treatment have inadvertently supported an exploration of alternative treatment modalities. These include, but are not limited to, play and creative arts approaches (88–92) and technology-mediated interventions (93–97). Such interventions are premised on being developmentally appropriate (e.g., play, visual artmaking) or innovative and culturally relevant (e.g., digital games). Unfortunately, findings on alternative modalities are likewise constrained by methodological limitations and inconsistent results on social outcomes (98–100). Nevertheless, the expansion of intervention research into these diverse domains reflects a growing view that addressing ASD social dysfunction requires more than targeting localized social deficits. It alludes to a tacit understanding of materiality, an engagement of the perpetual dialectic between autistic individuals and their situated environments, which warrants empirical clarification.

## Perspectives From Quality-of-Life, Cross-Cultural, and Patient-Outcome Studies

Quality-of-life (QoL) studies inquire autistic individuals' well-being, including social functioning, in everyday life and are less concerned with diagnostics and maladaptive traits. QoL measures may be domain-specific or -diverse and are often quantitative and based on parental or caregiver reports. Positive QoL in ASD has been associated with regular meaningful engagement (101) and adaptive social behaviors (102). Kuhlthau et al. found poorer social functioning but not school functioning in children with ASD. Differential findings could point to the influence of socializing opportunities and environmental adjustments made to cater to ASD needs. Parents of children with ASD have reported issues of lower school attendance, religious participation, and other organized activities (103). Yet, findings are limited to parent-reports, and it is uncertain whether these children perceived their reduced social participation poorly. Although bullying has also been reported, bullying is a societal issue that is confounded by factors beyond ASD-specific social deficits (104–106). Regardless, QoL studies help quantify or delineate some areas of real-life social functioning pertinent to ASD. While they are inherently bound by the measures used, variables investigated, and context under inquiry, QoL studies provide insights into the effects of ASD on actual non-clinical interpersonal situations.

Cross-cultural studies seek to reveal cultural impacts on ASD characterization or functioning. By implication, the dissimilarities and universal characteristics of ASD across different cultures or countries can be illuminated. Autistic children from Britain, Israel, and Sweden showed similar deficits in emotion recognition compared to neurotypical counterparts (107). Sipes et al. (108) found greater positive social skills, but no differences in hostile or inappropriate social behavior, in autistic children from the United States as compared to those from the United Kingdom. In another study, Taiwanese with ASD had more limited social participation than the Australian counterparts, with social participation being affected by gender, ASD symptom severity, and social anxiety (109). Sotgiu et al. (110) compared multiple psychosocial variables between Cuban and Italian children with and without ASD. They showed that Italian children had a wider social network, less frequent contact within network, and fewer multifunctional figures. No differences were found on mother-child attachment and cognitive or emotional competence. Apparently, most cross-cultural research has suspended the diagnostic assignment of valence to social functioning by establishing relationships among socially or culturally relevant factors.

Such studies often bring forth more questions than answers, as sociocultural factors are circumscribed and fail to serve as decisive explanations for variable ASD characteristics. Indeed, culture is a broad construct to be operationalized by selected measurable factors; overlaps among ASD-specific social behaviors, adaptive social functioning, and culturally situated social practices also remain fundamentally indistinguishable. There is some indication that ASD-specific social behaviors are normal in certain cultures. In Korea, cultural factors such as mainstream school structure may be befitting for autistic children, and ASD-related characteristics are attributable to mother-child interactions (111). ASD appears to be embedded within cultures, especially since cultures that emphasize communality regard autistic challenges as a collective problem of difference rather than disability (112). This normative perspective is further undergirded by social stigma research. Stigma has been found to predict greater camouflaging by autistic individuals to gain social acceptance and at the expense of the autistic person's psychological well-being (113–115). Depending on how social functioning is characterized, social functioning may be maladaptive from the perspective of the non-autistic world but not from that of the autistic individual.

Patient-outcome studies clarify the autistic individual's perspective on social functioning. Conclusions are mixed as individuals with ASD have reported both satisfactory and unsatisfactory social lives (116, 117). Consistent with stigma research, people living with ASD indicated poor social lives due to lack of others' understanding (118). In this interpretive phenomenological study, autistic individuals expressed that they valued familial-based or one-to-one support, desired more real-world practice, and did not find social communication interventions helpful. These subjective reports provide some indications of misalignment between autistic persons and others. However, misalignment has been indicated in terms of perceived individual challenges or intervention approaches rather than actual social behaviors. Parental stress has been attributed not to social interaction or communication deficits but to inadequate formal support, poor parental coping skills, autistic child's hyperactivity, challenging externalizing behaviors, and regulatory issues (119–121). Moreover, patient-outcome studies have been largely anecdotal in nature. Sample sizes are small, and participants evaluated are high functioning. It would be interesting to explore the lived experience involving a larger and more representative sample of both autistic and neurotypical individuals. By examining multiple stakeholders, the enduring perception of misalignment in social behaviors between autistic individuals and others can be better expounded.

## Discussion

The present interrogation reveals intricate perspectives on social functioning in ASD. Neuroscience and biology suggest that ASD is associated with structural or functional differences that could adversely impact social functioning and other clinical symptoms; yet interventions that target these anomalies yield mixed or modest interpersonal outcomes. Interventions are also highly variable in component and principle. Patient-outcome studies demonstrate how individuals with ASD find existing interventions unremarkable and could benefit from greater understanding among society. In addition, cross-cultural and QoL research purport a nuanced and pragmatic view of social functioning in ASD, indicating a need to examine ASD within its idiosyncratic sociocultural context. Even though the current literature alludes to a misalignment in social behaviors between autistic individuals and their community or society, it is uncertain where this disparity lies and how it is problematic. Consequently, evidence is not definitive to inform intervention. Research regarding ASD has come far, and much more is required.

### Expanded View in Basic Research

Neuroscience and biology continue to be key to informing ASD etiology, diagnosis, and treatment. Even though methodologies inquiring lived experiences seem compelling, participating individuals are often limited to those able to communicate their perspectives. This excludes many others on the spectrum with low intellectual and communicative abilities. Jaarsma and Welin (122) noted the inherent bias within the neurodiversity movement and argued for a narrow conception of neurodiversity. Specifically, high functioning ASD can be considered a difference that deserve equal rights and respect, but low functioning ASD remains a disability to warrant help. Therefore, basic research is essential for an inclusive understanding of the entire autism spectrum.

Investigation of intersubjective misalignment between autistic individuals and others will be critical insofar as ASD is characterized primarily by social deficits. As shown, basic research has assumed “social deficits” a priori, operationalizing them using singular proxy measures (e.g., joint attention as marker for social deficit) or a predetermined constellation of theoretically derived variables (e.g., social brain correlates). This implicit notion belies any difficulty related to social misalignment between autistic individuals and others. Is there a misalignment? If so, what is this misalignment and how does it produce interpersonal challenges? Thus, a more fundamental question appears to lie in defining and determining social dysfunction, rather than establishing a link between ASD and social dysfunction. This endeavor is challenging but not impossible. Measurement of discrepancy between parents' and autistic individuals' perceived social functioning has shown to be systematically meaningful (123). To expand beyond “calculating heritability estimates or conducting time-locked correlational analyses” of ASD deficits, Meek et al. (124) articulated the viability of a conceptual model that examines gene-environment correlations and their multiplier effect on social trajectories. Here, social functioning is viewed as process- and time-dependent, and its mechanisms studied through combined analyses of gene-environment correlations, social behaviors over time, as well as genetic, parenting, and school environment factors. Such inter-person, dynamic views could help discriminate misaligned social behaviors in context, whose impacts may or may not require intervention.

### Advancing Intervention Studies Through People-First Research

An expanded perspective is likewise necessary for the advancement of intervention research. Current treatment approaches are inadequate in improving socially relevant aspects of ASD and in appealing to autistic individuals. Therapeutic needs also differ depending on developmental stages or parental and familial goals (125). Therefore, multiple stakeholders should be considered in treatment design to address core interpersonal challenges. Malinverni et al. (126) explored a participatory design approach involving not only clinical experts but also children with ASD. Though preliminary, their approach was feasible, and intervention showed indications of improved social initiation in autistic children.

The neurodiversity movement recognizes affected individuals as experts of their own diagnoses and experiences. It also involves a perspective change from positivist (“what are social deficits”) to constructivist (“who judges what adaptive social functioning is”), which has fueled considerable debate (127). In *Autism in Translation* (128), Weisner discussed the applicability of psychological and medical anthropology research in integrating neurobiological and sociocultural perspectives of ASD. Nonetheless, research in this area is nascent. Interpellation of individuals with ASD will continue to rely on historically based medical models of codification that likely keep on evolving. Diagnoses remain crucial at present to safeguard interventions that support ASD needs. Importantly, people-first approaches need not preclude a positivist or objective examination of ASD features that would warrant clinical help. Even as a paradigm shift toward societal acceptance is apparent, a paradigm expansion seems more appropriate for current research and practice purposes.

Our review did not elucidate how restricted, repetitive behaviors central to ASD might contribute to social functioning. Ideally, these must be examined alongside “social deficits” to present a comprehensive view. We also did not exhaust anthropological findings that shed light on the phenomenological and meaning construction of ASD. Although we questioned the a priori treatment of social dysfunction in ASD across studies, this does not mean that respective conclusions are suspect. In fact, such a presumption is essential to render scientific inquiry possible. Ultimately, our review brings to fore a longstanding assumption associated with ASD research and practice. It is our intention that future research would explicate this presumption through greater interdisciplinary efforts or enabling an intersubjective locus of social functioning involving both autistic individuals and the non-autistic world.

## Data Availability Statement

The original contributions presented in the study are included in the article/supplementary material, further inquiries can be directed to the corresponding author.

## Author Contributions

XBL ideated, performed the literature search, and drafted the manuscript. CGL provided clinical expertise. TSL reviewed and critically revised the work. All authors contributed to the article and approved the submitted version.

## Conflict of Interest

The authors declare that the research was conducted in the absence of any commercial or financial relationships that could be construed as a potential conflict of interest.

## Publisher's Note

All claims expressed in this article are solely those of the authors and do not necessarily represent those of their affiliated organizations, or those of the publisher, the editors and the reviewers. Any product that may be evaluated in this article, or claim that may be made by its manufacturer, is not guaranteed or endorsed by the publisher.
